# Immunological repertoire linked to PSTPIP1-associated myeloid-related inflammatory (PAMI) syndrome

**DOI:** 10.1186/s12969-021-00617-y

**Published:** 2021-08-16

**Authors:** Leonardo Oliveira Mendonça, Maria Teresa Terreri, Fabiane Mitie Osaku, Samar Freschi Barros, Karen Francine Köhler, Alex Isidoro Prado, Myrthes Toledo Barros, Jorge Kalil, Fabio Fernandes Morato Castro

**Affiliations:** 1 Discipline of Clinical Immunology and Allergy, Universidade de São Paulo, School of Medicine, Rua Doutor Eneas de Carvalho Aguiar, 255, 8 andar, São Paulo, São Paulo 05403-000 Brazil; 2 Laboratory for Immunological Investigation (LIM-19), Heart Institute, University of São Paulo, São Paulo, Brazil; 3 Center for Rare and Immunological Disorders, Hospital 9 de Julho, São Paulo, Brazil; 4 Pediatric Rheumatology Department, Universidade Federal de São Paulo, School of Medicine, São Paulo, São Paulo Brazil; 5 Pediatric Rheumatology, Hospital Infantil Joana de Gusmão, Florianópolis, Santa Catarina Brazil

## Abstract

**Background:**

Mutations along PSTPIP1 gene are associated to two specific conditions, PAPA syndrome and PAMI syndrome, both autoinflammatory disorders associated to disturbances in cytoskeleton formation. Immunological aspects of PAMI syndrome has not yet been reported neither the clinical impact on therapeutical decisions.

**Methods:**

Clinical data of patients records were retrospectively accessed. Genomic DNA were extracted and sequenced following standard procedures. Peripheral lymphocytes were quantified in T, B e FOXP3 phenotypes.

**Results:**

We describe two related patients with PAMI syndrome harboring the usual E250K mutation. Anti-IL1 therapy could partially control the disease in the index patient. A broad spectrum of immunological effects as well as an aberrant expression of FOXP3 could be observed.

**Conclusions:**

Here we report two related brazilian patients with PAMI syndromes harboring the E250K mutation in PSTPIP1, their immunological aspects and the therapeutical response to canakinumab.

## Introduction

Proline-serine-threonine phosphatase-interacting protein 1 (PSTPIP1, also known as CD2BP1) is a cytoskeleton-associated adaptor protein that modulates T-cell activation, cytoskeletal organization and IL-1β release [1]. Mutations along the PSTPIP1 gene have been classically associated with PAPA syndrome (OMIM #604410), an autosomal dominant disorder [2]. However, specific heterozygous mutations (E250k and E257K) in this same gene were recently linked to another condition, namely PSTPIP1-associated myeloid-related inflammatory (PAMI) syndrome [3].

In addition to its known involvement in cytoskeletal organization, interaction between PSTPIP1 and the WASP protein lead to defective podosome formation and interfere with macrophage migration [4]. Additionally, gene MEFV, in which mutations have been associated with Familial Mediterranean fever (OMIM #249100), encodes the protein pyrin, which can alter the distribution of PSTPIP1 throughout the filamentous network [5]. Accordingly, PSTPIP1-related diseases are considered cytoskeleton-associated disorders.

PAMI syndrome is classified as a rare autoinflammatory condition clinically characterized by the early onset of multisystemic inflammation. Affected patients present with recurrent episodes of fever, osteoarticular symptoms, skin lesions, diffuse reticula-endothelial enlargement, anemia and thrombocytopenia. These symptoms are usually associated with high levels of acute reactant markers (CRP, ESR and SAA); hyperzincemia is also a consistent finding. At the time of this publication, just 20 cases had been reported in the literature [6].

Here we report two related Brazilian patients with PAMI syndrome, both harboring the E250K mutation, one of whom was treated with canakinumab (Ilaris). We further discuss our observations of wide-ranging immunological aspects regarding T, B and regulatory T cells.

## Methods

### Clinical data and genomic sequencing

Clinical data were retrieved from patient records after parents provided a signed term of consent. Genomic DNA was extracted from blood samples using a QIAamp® DNA Blood Maxi Kit (Qiagen®, Valencia, CA, USA) and peripheral blood mononuclear cells (PBMC) were obtained by density gradient centrifugation (d = 1.077 g/ml), then cryopreserved for later use. Exome sequencing was performed using a commercially available kit (Medelics Genomiks) and primers were designed to directly target exon 11 of the PSTPIP1 gene. Sanger sequencing was performed for genetic confirmation and familial segregation following standard procedures.

### Quantification of lymphocytic B, T and FOXP3 phenotypes by flow cytometry

PBMC of the index patient and the mother was collected and stored. Two samples from the index patient were obtained, one before canakinumab was started and without oral steroids and a second sample while on canakinumab and cyclosporine.

Peripheral blood mononuclear cells (PBMC) were stained with titrated mouse anti-human monoclonal antibodies (mAbs) (all from BD Biosciences), as following: anti-CD3 FITC or PerCp, anti- CD4 AmCyan or pacific Blue, anti-CD8 Pacific Blue or APCCy7 or PE-Cy7, anti-CD45RA PE-Cy7 or APC, anti-CCR7 (CD197) PerCp-Cy5.5, anti-CD27 APC or FITC, anti- CD16 PE, anti- CD56 PE, anti- CD19 PerCp, anti- CD20 PE-Cy7, anti- HLA-DR APC, anti- CD45 AmCyan, anti-CD62L PE, anti- TCRαβ FITC, anti- TCRγδ PE and anti-BB220 APC, according to manufacturer instructions. For Treg/CD28null panel, we stained the cell surface with anti-CD3 FITC, anti-CD4 AmCyan, anti-CD8 Pacific Blue, anti-CD25 PE-Cy7, anti-CD127 PE and CD28 APC. For intracellular staining of FOXP3 (PerCp-Cy5.5) cells were washed, fixed and permeabilized with Transcription Factor Buffer Set (BD Biosciences) immediately after surface staining, in accordance with the manufacturer’s instructions. Fluorescence minus one (FMO) control were set up for CD45RA, CD127 and Foxp3 markers.

Flow cytometry was performed in FACSCanto II (BD Biosciences) and the analyses were made in FlowJo 9.9.5 software (TreeStar Inc., San Carlos, CA, USA). After exclusion of cell doublets, sequential gating of PBMC was performed in the lymphocyte region. For T lymphocytes, after gate of CD3+ T cells, followed by discrimination of CD8+ and CD4+ markers, we analyzed the T lymphocyte naïve/memory subpopulations through boolean gates: T naive (CD45^+^CCR7^+^CD27^+^), T central memory (TCM) (CD45^−^CCR7^+^CD27^+^), T effector memory (TEM) (CD45^−^CCR7^−^CD27^+^) and T effector memory with RA re-expression (TEMRA) (CD45^+^CCR7^−^CD27^−^). To analyze the phenotypes CD3^+^ TCRaß CD4^−^ CD8^−^, CD3^+^ HLA-DR^+^ and CD3^+^ B220^+^, the cells were gated on CD3^+^ region, after exclusion of doublets and death cells. The phenotypes CD27^+^ was analyzed in B lymphocyte region (CD20^+^ cells). Lymphocyte subsets absolute counts were calculated using the percentages obtained in flow cytometry. The subset percentages analyzed were referred to total lymphocyte counts for T and B cells.

For Treg panel, we analized the following subpopulations within the CD4+ subgroup: TREG (CD25^high^, CD127-, FOXP3+), TCD4/CD25+ (CD25+, CD127-, FOXP3+), TCD4 FOXP3+, TCD4 FOXP3+ CD28^null^ and TCD4 CD28^null^. Within the CD8+ subgroup, we analyzed TCD8 FOXP3+, TCD8 FOXP3+ CD28^null^ and TCD8 CD28^null^. The T cell subsets analyzed are expressed as percentages, referring to the total number of viable TCD4 and TCD8 lymphocytes.

T and B cell population results were compared to reference ranges from Brazilian subjects published in the literature. Treg cell values were compared to a patient harboring a TRNT1 mutation (a complex autoinflammatory condition considered to have normal Treg expression) and to a patient harboring a FOXP3 mutation (considered to have low expression of Treg).

## ^l^Results

### Case report

A 4-year-old girl in southern Brazil, born to non-consanguineous parents of German-Italian descent, came to our attention due to the suspicion of an inborn error of immunity. Her parents reported high fever in association with diarrhea, paleness and mucosal bleeding in her first month of life. She was first hospitalized at that time due to a severe and progressive deterioration in her clinical picture. The patient appeared severely dehydrated, with profoundly low levels of hemoglobin (6.6 mg/dL - reference range 11.5–15.5 g/dL), neutropenia (548 cells - reference range 2500 - 7500/mm^3^), thrombocytopenia (20,000 platelets - reference range 150,000-400,000) and high levels of CRP (57.6 mg/dL - reference range < 0.5 mg/dL). The patient presented high cytomegalovirus viraemia in peripheral blood. Clinical remission was observed following prolonged intravenous administration of ganciclovir at standard doses.

**Fig. 1:**
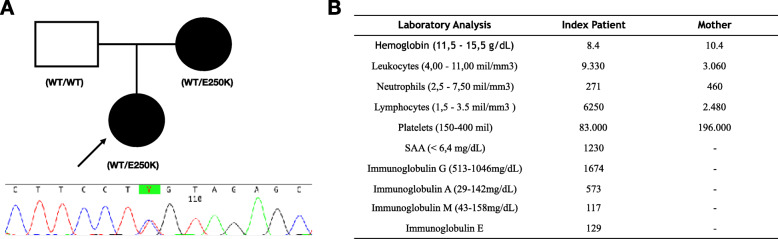
Genetic sequencing and baseline laboratory analysis of patients harboring the E250K mutation in gene PSTIPIP1. A) Family pedigree and electropherograms demonstrating an autosomal dominant pattern of inheritance. B) Baseline laboratory analysis of the index patient and her mother, evidencing anemia, neutropenia in both patients and thrombocytopenia in the index patient

**Fig. 2:**
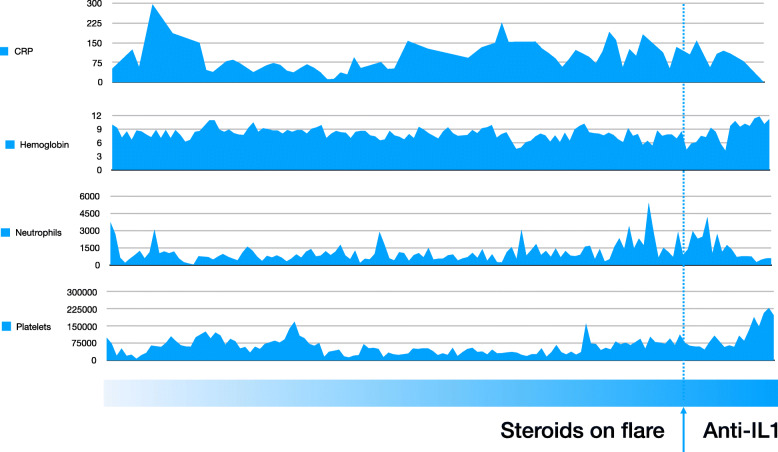
Four-year follow-up of laboratory parameters in the index patient, including response to anti-IL1B therapy. Systemic inflammation associated with marked hematological involvement is observed, with high levels of C-reactive protein (CRP), anemia, neutropenia and thrombocytopenia. Dashed lines indicate the onset of anti-IL1B (canakinumab)

Monthly episodes of fever, lasting from 2 to 3 days, were noted in association with intermittent and non-specific macular skin rash. Persistent splenomegaly and severe and refractory anemia, thrombocytopenia and neutropenia, as well as high levels of acute reactant markers (ESR, CRP and SAA) were consistent findings upon flares. Several hospitalizations ensued, entailing blood transfusions and the intravenous administration of broad-spectrum antibiotics to treat undefined systemic infections. During this period, a failure to thrive could also be observed. At the age of two years, an empiric dose of corticosteroids (1 mg/kg) was introduced, resulting in partial resolution of clinical manifestations. Steroids resulted in less frequent hospitalization, extended the intervals between febrile episodes, and the amelioration of diarrhea, skin rash and arthritis. However, the patient presented failure to no less than two steroid sparing agents: IVIG and colchicine. Consequently, steroids were administered on demand during flares at a dose of 1 mg/kg for 5–7 days. On two occasions serum zinc levels were evaluated in the index patient, returning results within normal range. Of note, the patient’s mother also complained of recurrent fever and thrombocytopenia since early in life, yet remained asymptomatic throughout adulthood. Therefore, anti-IL1 (canakinumab) at a dose of 2 mg/kg/month was initiated in the index patient and progressively titered until reaching a dose of 4 mg/kg/month. Following the onset of anti-IL1 therapy, persistent clinical amelioration was observed in addition to the normalization of platelet and hemoglobin levels, yet neutrophil counts remained low. While systemic inflammatory flares were still noted following anti-IL1 therapy, these were less severe and some resolved spontaneously (Fig. 2). After two years of follow-up after anti-IL1 was initiated no other hospitalization could be observed. Her mother was not treated and is currently being regularly followed on an outpatient basis. Curiously despite the mother is completely asymptomatic high levels of acute reactant markers could be observed (Fig. 1B).

### Genomic sequencing

Upon the suspicion of an inborn error of immunity a commercially available exome sequencing was requested. A previously reported pathogenic mutation in PSTPIP1, c.748G > A, p.Glu250Lys, was confirmed by Sanger sequencing. Segregation analysis confirmed that the mother harbored the same mutation (Fig. 1A).

### Peripheral lymphocyte repertoire

We observed that both, index patient and the mother expressed high peripheral expression of T CD8 cells, low peripheral expression of T CD4 and normal B (CD19) cells, and very low levels of nk cells (Table 1). The index patient presented with high levels of immunoglobulins A, G and normal levels of immunoglobulin M (Fig. 1B). High levels of double-negative T cells and B220 cells were also noted in both, the mother and the index patient (Table 1).

**Table 1:** Immunological repertoire obtained prior to the onset of anti-IL1B therapy evidencing multiple immune defects (relevant alterations shown in bold)

Immunological Profile	Index Patient Before anti-IL1 (%)	Age Matched Reference Range	Mother untreated (%)	Age Matched Reference Range
CD3+	38,9	66.2 (57.1-72.7)	81	75.1 56.8-84.1
CD4+	22,6	37.7 (27.7-46.3)	36,5	40.7 26.9-55.5
CD8+	**70,7**	21.9 (15.7-33.8)	**58**	27.2 13.3-41.5
CD4+ naive	**23,9**	70.30 (46.14-84.40)	**16,6**	32,7 (20,9-49,1)
CD4+ TCM	**10,8**	26.40 (13.88-48.12)	**32**	33 (20,8-45,6)
CD4+ TEM	5,41	2,8 (0.94-6.46)	11,4	25,1 (13,9-33,6)
CD4+ TEMRA	1,59	0.2 (0.00-1.36)	1,4	4,9 (2-10,3)
CD19+	21,3	19,3 (13.3-26.7)	9,16	12 (5.9-20.6)
CD3-CD16+CD56+(NK)	**1,2**	7,3 (7,8-16,1)	**3,34**	8.2 (2.8-24.1)
CD3+HLA-DR+	43,5	NR	48,7	NR
CD20+CD27+(LB mem)	27,9	NR	29,1	NR
CD3+TCRabCD4-CD8-	**2,83**	Total Lymphocytes (<1,5%)	**2,01**	Total Lymphocytes (<1,5%)
	7,54	CD3+(<2,5%)	NM	CD3+(<2,5%)
CD3+B220+	31,7	Total Lymphocytes (NR)	15,8	Total Lymphocytes (NR)

We identified lower FOXP3 expression in T CD4 cells, especially in cells CD127+ (cytokine IL-7) that ameliorated after anti-IL1 therapy. We further observed high CD28^null^ expression in T-CD4 and T-CD8 cells, as well as FOXP3 expression dependent on CD28^null^ only in T-CD4 cells (Table 2). Conversely, high levels of FOXP3 in T-CD8 cells had been observed in the index patient that diminished after anti-IL1 therapy. All FOXP3 expressions were compared to the mother, as an untreated PAMI patient and to an IPEX-FOXP3 patient, considered to have very low expression of FOXP3 (Fig. 2).

**Table 2:** FOXP3 expression prior and after the initiation of anti-IL1 therapy (A and B) in the index patient and FOXP3 expression in the mother (untreated). Notable increased expression of FOXP3 can be observed after anti-IL1 therapy was initiated

Immunological Profile	Index Patient – A (%)	Index Patient – B (%)	Mother (%)	IPEX Patient (%)
CD4+CD25+CD127-FOXP3	12,4	**58,2**	77,9	9,18
CD4+CD28null	37,6	0,92	1,25	1,58
CD4+CD28null FOXP3+	21,5	**33,2**	19,4	0,00
CD4+FOXP3+	63,5	14,7	14,4	0,00
CD4+CD25+FOXP3+	33,5	**51,4**	47,5	0,74
CD8+FOXP3+	8,44	11,5	9,3	0,00
CD8+CD28null	87,9	16,7	10,7	16,20
CD8+CD28null FOXP3+	7,39	14,9	7,32	1,39

## Discussion

Here we describe a case of two related patients harboring the typical E250K mutation in PSTIPIP1 who present classical signs of PAMI syndrome [1]. While other patients typically present hyperzincemia and hypercalprotectinemia, these were not seen in the young patient. This finding should be considered highly relevant, as patients lacking these characteristics must also be considered eligible for PSTIPIP1 genetic screening [3, 5].

As indicated by previous reports, no effective therapy exists for affected patients. Anakinra, an anti-IL1 receptor agonist, was demonstrated to partially control disease activity. The present report describes the first use of canakinumab, an anti-IL1B monoclonal antibodyB, in the treatment of this syndrome. Despite its significant control over the frequency and intensity of disease flares, canakinumab does not seem to be as thoroughly effective as anakinra in patients with PAMI syndrome. Bone marrow transplantation has been reported in just one patient refractory to a broad spectrum of immunosuppressants [5].

The immunological aspects of PAMI syndrome have not yet been reported. As F-actin is essential for immunological synapse (IS) formation [4, 7], it follows that the strongly diminished actin formation in patients harboring pathogenic mutations in PSTIPIP1 may lead to a wide range of T cell-related immune defects, as observed in the index patient (Table 1). Patients harboring other mutations along PSTIPIP1 (e.g. R228C and T274M) who present a clinical phenotype of common variable immune deficiency without autoinflammation also exhibit similar T cell defects as those observed in our patient [7]. Since PSTIPIP1 is highly expressed in T cells rather than B cells, this may serve to explain the normal levels and functioning of B cells observed in our patient. Signs associated with T cell-related “immunodeficiency” may also explain the severe clinical course of CMV infection observed in the early life of our patient.

A subset of CD4^+^ T cells, Tregs are capable of limiting effector CD4^+^ T cells and suppressing immune-mediated inflammations. PSTIPIP1 induces the activation of the so-called “pyroptosome,” which leads to cell death and the release of cytokines, such as IL-1β, via the NLRP3 inflammasome that negatively regulates Treg cell differentiation [1, 7, 8].. Therefore, it is possible that NLRP3 is involved in T cell regulation via FOXP3 expression (Table 1) in association with the high levels of double-negative T cells (Table 1) observed herein may be the result of non-canonical activation driven by PSTIPIP1. Our findings may shed light on a new pathway of FOXP3 activation mediated by PSTIPIP1 in PAMI syndrome, which could entail therapeutic implications.

## Conclusions

PAMI syndrome is a recently categorized autoinflammatory disorder clinically characterized by hematological involvement and recurrent episodes of autoinflammation. Here we describe two related patients with PAMI syndrome, harboring the same typical E205k mutation. We report for the first time a severe CMV infection as the first manifestation of PAMI syndrome. Our observations of T cell defects may help elucidate host predisposition to particular infections, such as cytomegalovirus here reported. The role of TReg cells in PAMI syndrome, as well the participation of PSTIPIP1 in Treg cell activation and differentiation require further evaluation.

## Data Availability

The datasets used and/or analyzed during the current study are available from the corresponding author on reasonable request.

## Abbreviations

IVIG: Intravenous Immunoglobulin
SIFD: Syderoblastic anemia, recurrent fevers and development delay Syndrome
PSTPIP1: Proline-Serine-Threonine Phosphatase Interacting Protein 1
CD2BP1: CD2-binding protein 1
PAPA: Pyogenic Arthritis, pyoderma gangrenous and acne
WASP: Wiskott–Aldrich Syndrome protein
MEFV: Mediterranean Fever
CRP: C-reactive Protein
ESR: erythrocyte sedimentation rate
SAA: Serum Amyloid
FOXP3: Forkhead Box 3
CD: Cluster Differentiation
TREG: T-regulatory cells
TRNT1: tRNA-nucleotidyltransferase 1
